# Significance of Tool Coating Properties and Compacted Graphite Iron Microstructure for Tool Selection in Extreme Machining

**DOI:** 10.3390/nano15020130

**Published:** 2025-01-16

**Authors:** Anna Maria Esposito, Qianxi He, Jose M. DePaiva, Stephen C. Veldhuis

**Affiliations:** 1 McMaster Manufacturing Research Institute, McMaster University, Hamilton, ON L8P 0A6, Canada; 2 Department of Mechanical Engineering, McGill University, Montreal, QC H3A 0C3, Canada

**Keywords:** compacted graphite iron (CGI), tool wear, machinability, AlTiN-based coatings, tool selection

## Abstract

This study aims to determine the extent to which coating composition and workpiece properties impact machinability and tool selection when turning Compacted Graphite Iron (CGI) under extreme roughing conditions. Two CGI workpieces, differing in pearlite content and graphite nodularity, were machined at a cutting speed of 180 m/min, feed rate of 0.18 mm/rev, and depth of cut of 3 mm. To assess the impact of tool properties across a wide range of commercially available tools, four diverse multilayered cemented carbide tools were evaluated: Tool A and Tool B with a thin AlTiSiN PVD coating, Tool C with a thick Al_2_O_3_-TiCN CVD coating, and Tool D with a thin Al_2_O_3_-TiC PVD coating. The machinability of CGI and wear mechanisms were analyzed using pre-cutting characterization, in-process optical microscopy, and post-test SEM analysis. The results revealed that CGI microstructural variations only affected tool life for Tool A, with a 110% increase in tool life between machining CGI Grade B and Grade A, but that the effects were negligible for all other tools. Tool C had a 250% and 70% longer tool life compared to the next best performance (Tool A) for CGI Grade A and CGI Grade B, respectively. With its thick CVD-coating, Tool C consistently outperformed the others due to its superior protection of the flank face and cutting edge under high-stress conditions. The cutting-induced stresses played a more significant role in the tool wear process than minor differences in workpiece microstructure or tool properties, and a thick CVD coating was most effective in addressing the tool wear effects for the extreme roughing conditions. However, differences in tool life for Tool A showed that tool behavior cannot be predicted based on a single system parameter, even for extreme conditions. Instead, tool properties, workpiece properties, cutting conditions, and their interactions should be considered collectively to evaluate the extent that an individual parameter impacts machinability. This research demonstrates that a comprehensive approach such as this can allow for more effective tool selection and thus lead to significant cost savings and more efficient manufacturing operations.

## 1. Introduction

Compacted Graphite Iron (CGI) is a form of cast iron used increasingly in the automotive industry to build diesel engine blocks due to its superior physical and mechanical properties over Gray Cast Iron (FGI) and better machinability over Ductile Cast Iron (NGI). All three classes of cast iron consist of pearlite and ferrite with graphite inclusions. In FGI, graphite is in lamellar form, resulting in a relatively brittle and easy-to-machine material. NGI is a high-strength, ductile material with spherical graphite nodules that significantly increase its toughness but decrease machinability. CGI exhibits intermediate properties with vermicular graphite appearing as randomly oriented worm-like structures. Due to the interconnected nature of the graphite, there are fewer fracture planes in CGI compared to FGI [1,2]. Furthermore, the vermicular graphite allows for greater thermal conductivity [2]. CGI therefore exhibits higher toughness and improved thermal properties over FGI, but better machinability than NGI. This leads to CGI being an ideal sustainable solution as it allows for the manufacturing of engines with high power output capabilities and excellent thermal conductivity, thus reducing energy consumption [2,3,4,5,6]. Despite its better machinability compared to NGI, the machinability of CGI is considered poor and is the main factor limiting its use [1,2,3,5,7]. As such, selecting the appropriate tool to machine CGI is critical to ensuring its viability as a sustainable solution.

The machinability of CGI can vary between workpieces due to differences in microstructure and composition, which must be considered in the tool selection process. Typical ranges and effects of this variability are outlined by Dawson [2]. Most of the literature focuses on the lack of MnS tribolayer formation in CGI as a side-effect of increased nodularity, both within the CGI classification and compared to FGI [1,2,3,8]. In a study comparing FGI and various grades of CGI, Gastel [8] also touches on the machinability challenges with increased pearlite content but, overall, there is minimal exploration of other implications of microstructural differences. Furthermore, there is little research investigating the optimization of tool selection specific to the microstructural differences of each workpiece sample.

Another factor that must be considered when selecting the appropriate tool for a cutting process is tool properties. Most of the literature is focused on developing hard, PVD-coated cemented carbide tooling to address CGI machinability issues [6,9]. When machining CGI, the industry standard is to use PVD-deposited AlTiN coatings [4,8,9,10,11]. Tooptong [10] states that the Ti component is hard and protects the tool against abrasion, while the Al component oxidizes to form a thermally stable Al_2_O_3_ layer. It is found that thick, hard coatings work best to mitigate wear because they reduce substrate exposure and can increase tool life proportionally to the increase in thickness [6,7,10]. De Paiva explored the effect of adding Cr to thin TiAlN coatings when drilling CGI and found that at low speeds, the Cr-based coatings outperformed the others, but at high speeds, all tools failed rapidly, and the effects of Cr were negligible [6]. Some emerging research investigates the effect of adding Si to AlTiN coatings when machining hard-to-cut materials [11,12]. Zhang compared various multilayered AlTiSiN tools and found that in addition to superior thermal properties, Si-containing coatings with a thin TiSiN top layer reduced adhesion wear due to a high hardness [12]. Given the beneficial effects of Si-containing coatings when machining other hard-to-cut materials, it is worth exploring whether similar outcomes are seen in applications with CGI.

From the literature, it is evident that different coatings address different wear mechanisms. Therefore, it is important to understand the dominant wear mechanism to select an appropriate tool. Guo [3] and Gastel [8] state that CGI is highly susceptible to thermally activated wear mechanisms due to the high temperatures incurred by large stresses. Others claim that CGI is most sensitive to abrasion and adhesion [4,6,7,10,13,14]. Both [3] and [4] refer to the increased ductility of CGI compared to FGI, with Guo noting that increased ductility leads to a longer chip–tool contact length, which increases temperatures [3], and with Sandoval stating that the increased ductility of CGI leads to a higher adhesive wear rate [4]. The dominant wear mechanism also depends on the cutting conditions and tool–workpiece interactions. For example, researchers citing sensitivity to adhesion and abrasion use moderate cutting speeds, at around 250 m/min [4,7,10], while researchers citing sensitivity to thermally activated wear mechanisms use higher cutting speeds, at 400–800 m/min [3,8]. Other researchers use different cutting processes altogether, such as Silva [13] and De Paiva [6] who studied the machinability of CGI during drilling. The cutting conditions and tool combinations used in the literature are summarized in Table 1. From the summary, it can be seen that tool wear and the machinability of CGI are primarily studied under moderate conditions. If one parameter is increased significantly, the cutting conditions are compensated by reducing other parameters, such as Abele’s study where high cutting speeds are compensated with a shallow depth of cut [1]. However, the tool wear mechanism and tool performance are not typically explored under extreme conditions. Furthermore, recalling De Paiva’s observation that the Cr content in tool coatings lost any advantage over typical AlTiN coatings at high speeds [6], it raises the question of whether improvements in tool performance obtained at moderate conditions remain significant at extreme conditions.

The primary goal of this study is to evaluate the significance of tool and workpiece properties to the tool selection process while machining CGI under extreme conditions in the form of a large depth of cut, high feed, and moderate speed. To address the gaps in the literature concerning the effects of CGI workpiece properties on the tool selection process, the machinability and wear behavior of two grades of CGI will be compared using commercially available carbide tools. Si-containing AlTiN coatings will be selected to determine whether the improved performance observed for other hard-to-cut materials is also observed when machining CGI. By exploring these novel considerations in the tool selection process using a material properties approach, this research aims to provide insight into how CGI interacts with cutting tools so that future innovations can be more effectively implemented when subjected to large stresses.

## 2. Experimentation

### 2.1. Workpiece Characterization

Two grades of CGI were tested in this study. Cylindrical workpieces were initially 134 mm in diameter and 150 mm long. Workpiece samples were polished and etched for microstructural analysis. A 2% nital reagent was used with a 1–3 s exposure time. Differences in graphite shape and pearlite content between the two grades were then measured on a digital optical microscope (Keyence, Osaka, Japan). Workpiece hardness was measured using the Vickers Hardness Test, which was then converted to Brinell Hardness values.

### 2.2. Tool Characterization

Four commercially available SNMN120408 tungsten carbide tools were used in this study. Tool A and Tool B were reported to have a thin, multilayered PVD AlTiSiN coating; Tool C was reported to have a thick, multilayered CVD Al_2_O_3_-TiCN coating; and Tool D was reported to have a thin, multilayered PVD Al_2_O_3_-TiC coating.

Geometric features were evaluated using the optical microscope (Keyence, Osaka, Japan) and 3D optical surface measurement system (Alicona, Raaba, Austria). The coating thicknesses were measured using SEM and EDS electron microscopy (JEOL 6610LV, Tokyo, Japan). This was also used for cutting tool chemical composition, microstructure, and morphology measurements.

Finally, several measurements were made to quantify the mechanical properties and tribology of each tool. The Palmqvist toughness test was used in accordance with ISO 28079 [15] to measure the fracture toughness of each tool. A load P of 30 kgf or 294 N was applied and crack lengths L were measured from crack tip to indentation corner. Then, the toughness W [N/μm]  was calculated according to W=P/L.

The hardness and elastic modulus of the coatings were measured using a nano-indentation tester (Anton Paar, Graz, Austria) to assess their micro-mechanical characteristics. A Berkovich diamond indenter was used for the indentation. A 20 mN load was applied. In order to prevent the substrate effect, this load was chosen to ensure that the indentation depth achieved was less than 1/10 of the coating thickness. Each sample underwent a total of 40 indentations.

The adhesion of the coatings to the WC substrate was examined through scratch tests conducted by a scratch tester (Anton Paar, Graz, Austria). This was carried out using a total track distance of 3 mm and an increasing load ranging from 0.5 to 100 N. A 200 μm radius Rockwell diamond indenter was employed.

### 2.3. Cutting Tests

Cutting tests were performed on a CNC Lathe (Boehringer, Ingelheim am Rhein, Germany) under flood coolant conditions with a 6% Casterol Hysol MBSO coolant concentration. Tool life tests were performed with a cutting speed of 180 m/min, feed rate of 0.18 mm/rev, and depth of cut of 3 mm. An additional test was carried out for CGI Grade A at an increased speed of 250 m/min. Two trials were performed for each test.

Tool life measurements were made in accordance with ISO 3685 [16] on the Keyence optical microscope. Flank wear was measured after every ~150 mm pass until end of life at an average flank wear width of 300 μm, as per ISO 3685 [16].

Cutting forces were measured using a Dynamometer (Kistler 9129AA, Winterthur, Switzerland). Because of the extreme cutting conditions, the forces exceeded the acceptable range for the Dynamometer. Therefore, additional tests were carried out by measuring the average Pass 1 cutting forces with a reduced depth of cut of 1 mm. Three trials were performed for each workpiece–tool combination.

### 2.4. Chip Analysis

A chip analysis was conducted to assess the tribological properties and performances of each tool. Chips were collected after the first cutting pass and analyzed on the SEM and EDS electron microscope. Shear bands, curling, and slipping patterns were examined as part of this test.

### 2.5. Wear Analysis

To observe the gradual tool wear progression, in-process wear images were taken after every pass on the Keyence microscope. Images were taken of the tool flank and rake surfaces, as well as the cutting edge.

Upon tool failure, SEM and EDS microscopy were performed to explore evidence of different wear mechanisms. The EDS chemical composition data were used to identify areas of substrate exposure, evidence of oxidation and corrosion, and adhered material.

## 3. Results and Discussion

### 3.1. Workpiece Characterization

**Figure 1:**
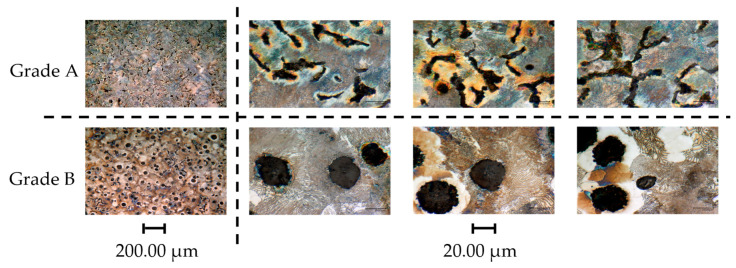
CGI workpiece microstructure at two different magnifications.

Optical analysis of etched workpiece samples showed significant differences in graphite shape between CGI Grade A and Grade B. In Grade A, graphite was primarily in nodular lamellae, whereas in Grade B, graphite appeared mainly in nodules, as seen by the black regions in Figure 1. For both workpieces, the microstructure was nearly entirely pearlitic, with negligible differences noted between grades.

The average Brinell hardness value for CGI Grade A was 224 ± 25 BHB and the average hardness for Grade B was 248 ± 38 BHB. Measurements were taken with various degrees of overlap with graphite to obtain an average value, but this also led to a large range of measurements. Given the large spread of the data, the differences in hardness values between Grade A and Grade B were statistically insignificant. This is expected because despite differences in graphite nodularity, the pearlite content between grades was effectively identical. Therefore, any differences in machinability were due to the effects of graphite nodularity. As discussed by Dawson [2], these effects include the tendency for increased graphite nodularity to result in greater ductility and tensile strength, which reduces machinability [2]. A study by da Silva [13] supports Dawson’s observations. In this study, three grades of CGI with varying degrees of graphite nodularity were investigated, with the lowest-nodularity CGI exhibiting the lowest hardness and longest tool life, and the highest-nodularity CGI exhibiting the highest hardness and shortest tool life [13]. Therefore, Grade B was expected to have a poorer machinability than Grade A.

### 3.2. Tool Characterization

The geometric properties of the tools are summarized in Table 2. Tool C’s large cutting-edge radius could be attributed to its thick coating, so the detrimental effects from the radius may have been offset. This does not hold true for Tool B, which had both a large cutting-edge radius and thin coating. All tools showed comparable roughness values, except Tool D, whose roughness was lower. High surface roughness can result in poor friction performance, but because the difference in roughness values was relatively minimal and because friction performance also depends on other properties, such as tribolayer formation, tool roughness may not be as significant as other properties in this application [17].

**Figure 2:**
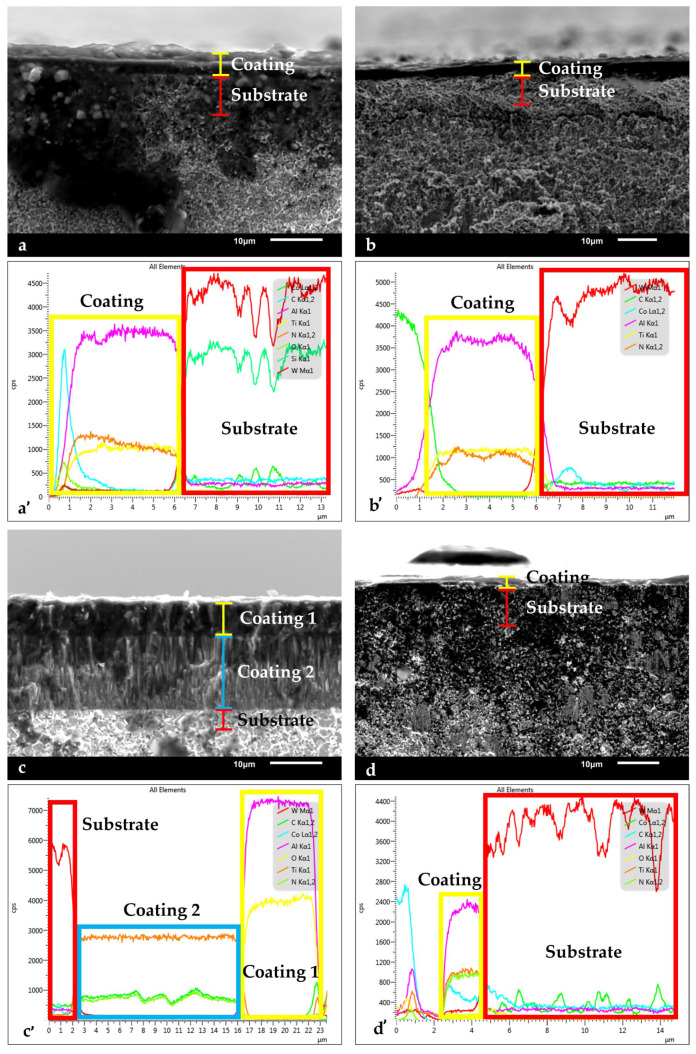
SEM/EDS coating and substrate cross-section for (**a**,**a’**) Tool A; (**b**,**b’**) Tool B; (**c**,**c’**) Tool C; and (**d**,**d’**) Tool D.

Figure 2 shows the coating cross-section microstructures. In Figure 2c, the columnar structure of the coatings on Tool C can clearly be seen. This phenomenon occurs during the nucleation process, where grains with energetically favorable orientations tend to grow both upwards and outwards. This preferential growth leads to the development of a strong crystallographic texture within the material, ultimately resulting in columnar growth structures [18,19]. Similar structural features are commonly observed in AlTiN-based coatings [20,21]. Despite not showing clearly defined layers, Tools A, B, and D may still have been multilayered. The tools may have been nano-structured multilayered tools, such as those studied by Skordaris [22], in which case the layers would not be identifiable on the SEM scale used. Furthermore, for AlTiN-based coatings with Si as an alloying element, columnar grain growth is inhibited, and grain size is refined due to the presence of Si [11,12]. This can result in an amorphous grain structure, which may have been the case for Tools A and B given that they had Si-containing coatings, as specified by the manufacturers.

The EDS data suggest that the coatings in Tools A, B, and D consisted of many thin coatings, as the amount of each constituent in the coating appeared approximately constant throughout the coating thickness. As a result, it can be assumed that the coatings simultaneously exhibited the beneficial properties of each component of the coating, such as the abrasion resistance of Ti compounds and thermal stability of Al_2_O_3_. This assumption does not hold for Tool C, as the thick outer Al_2_O_3_ layer must be worn before the inner TiCN coating is exposed. Therefore, the TiCN layer was protected from any thermally activated process long into the tool’s life. One feature to note is the high amount of carbon around the tool surface for Tool A. One of the tools studied by Zhang had a layer of amorphous carbon near the tool surface, which the author stated mitigated adhesion wear due to the self-lubricating properties of the carbon [12]. A similar resistance to adhesion was therefore expected for Tool A.

**Figure 3:**
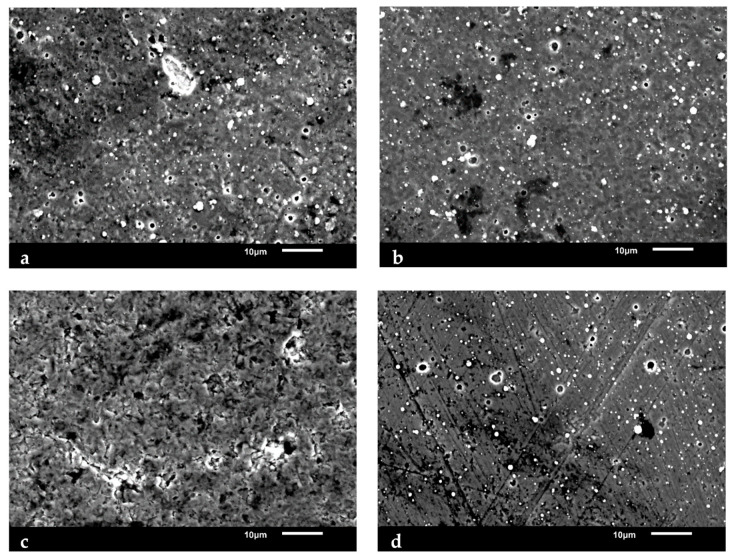
SEM tool surface images for (**a**) Tool A; (**b**) Tool B; (**c**) Tool C; and (**d**) Tool D.

Figure 3 shows the tool top surfaces. It is evident that the top layer of each coating contained droplet defects of different sizes and shapes. Coatings B and D exhibited nearly homogeneous porosities, whereas coatings A and C had some irregularly shaped droplets and porosities. Arcs can emit micro-droplets during coating deposition, which are then integrated into a coating as it develops [23]. Previous researchers have also noted that the coating’s mechanical properties, including hardness and adherence to the substrate, may have been impacted by the appearance of the flaws and porosities [21,24]. Surface irregularities, such as those from droplets, can enable crack propagation, which serves to decrease a tool’s wear resistance [24]. Furthermore, surface irregularities typically result in a higher surface roughness, which introduces additional frictional stresses while machining and thus can accelerate tool failure [24]. In addition to the surface flaws from droplets, Tool C also appeared to have a scale-like surface topology, which could further impact its mechanical properties. Tool D may not have exhibited flaws from droplets and non-uniform porosity, but Figure 3d shows that its surface was covered in scratches. As such, its mechanical properties may have also been affected by surface defects.

**Figure 4:**
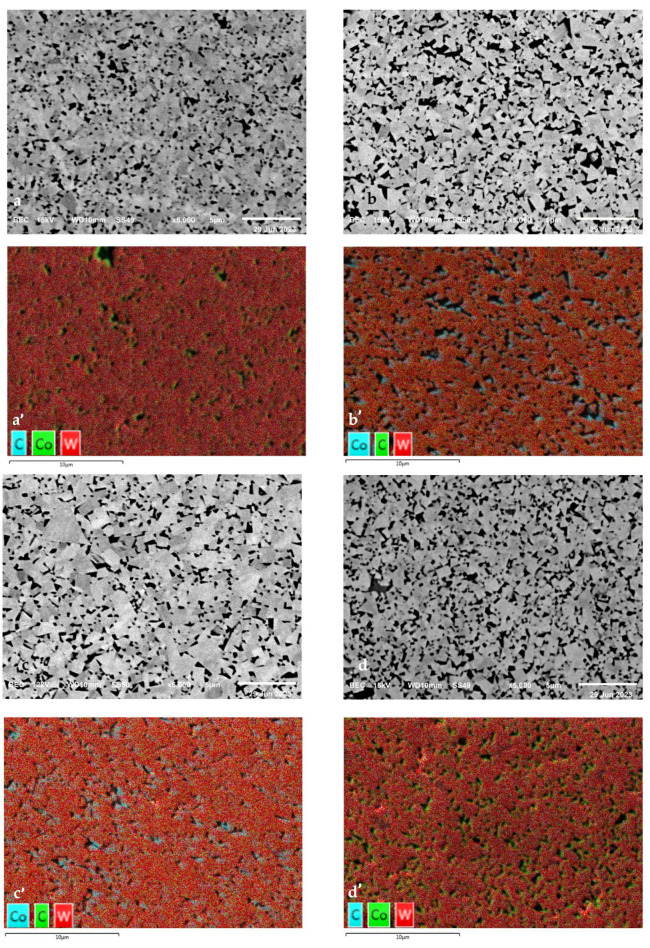
SEM/EDS substrate microstructure for (**a**,**a’**) Tool A; (**b**,**b’**) Tool B; (**c**,**c’**) Tool C; and (**d**,**d’**) Tool D.

Figure 4 shows the substrate microstructures for each tool. The dark phases indicate the Co binder, and the gray phases indicate the WC grains. The different shades of gray within the WC grains are due to orientation effects. The interaction between WC grain size and Co binder content are most critical to determining substrate properties [25]. A fine grain size and low binder content result in a high tool hardness, a fine grain size and a high binder content result in a high edge line toughness, a coarse grain size and a low binder content result in good thermal properties, and a coarse grain size and high binder content result in high bulk toughness [25]. Tools A, B, and D had a medium grain size, while Tool C had a coarse grain size. From EDS data, Tools A and C had the lowest Co content at approximately 3 wt%. Tools B and D had a Co content of approximately 10 wt%. Therefore, Tool A could be expected to have the highest substrate hardness but low toughness, Tools B and D could be expected to have the highest edge line toughness but poor thermal properties, and Tool C could be expected to have the best thermal properties but poor edge line toughness. Note that for Tool C, the coating comprised 50% of the total tool thickness compared to only 5% for the other tools. As such, most of the cutting load for Tool C was sustained by the coating, leading to a lower significance for substrate properties.

The results for the analysis of coating mechanical properties of the tools tested are summarized in Table 3. The hardness and toughness of the four coatings all followed the same trend, with Tool A exhibiting the lowest values and Tool D the highest. However, Tool B had the highest H/E value, which was caused by its high hardness and moderate elastic modulus. A material’s H/E value relates to its ability to dissipate energy. Tool B’s high H/E value suggests that it had a higher Si content than Tool A. As the Si content increases, so too does the grain refinement, and therefore hardness [12]. A high hardness can provide wear resistance but can also increase cutting temperatures and may affect coating adhesion.

The scratch tests explored the adhesion properties of the coating to the substrate. The first critical load, Lc1, indicates cohesive failure of the coating, while the second critical load, Lc2, indicates adhesive failure to the substrate. The results of the scratch test show that Tools A and D had similar Lc1 scores, while Tool B had a higher Lc1 score, and Tool C had a significantly lower Lc1 score. Tool B also had the highest Lc2 score, with Tools C and D exhibiting moderate scores and Tool A exhibiting the lowest.

**Figure 5:**
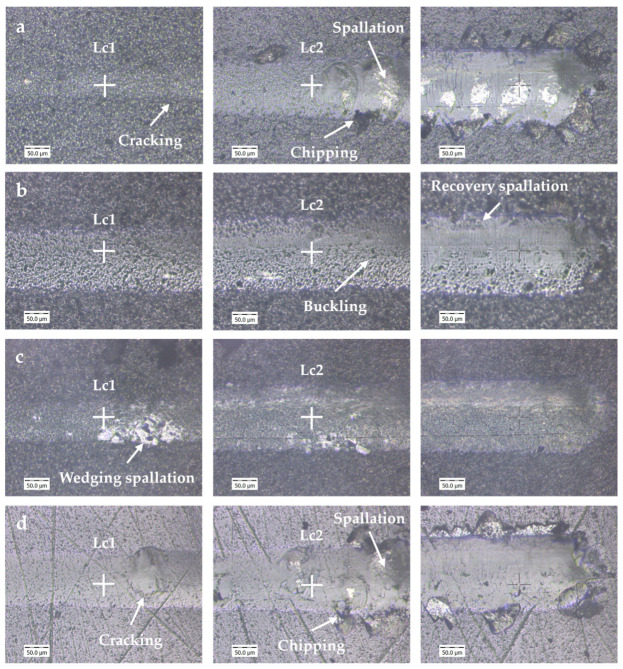
Scratch test images for (**a**) Tool A; (**b**) Tool B; (**c**) Tool C; and (**d**) Tool D.

At Lc1, Tools A and D showed evidence of cracking, as seen in Figure 5a,d, followed by gross spallation and chipping at Lc2. For Tool A, the cracks appeared laterally and were minimal, and spallation and chipping occurred both for the coating and the substrate, which is characteristic of a brittle coating and brittle substrate [26]. For Tool D, the cracks appeared Hertzian, and chipping of the substrate was minimal, which is characteristic of a brittle coating and ductile substrate [26]. Given the rapid gross spallation of Tools A and D, it can be concluded that these tools had very poor adhesion to the substrate, particularly Tool A [26]. For Tool D, this can be explained by the phenomenon that when two unlike surfaces are in contact, such as with a brittle coating and ductile substrate, the adhesive bonds between them are lower [27]. For Tool A, the poor coating adhesion may have been a result of its coating composition. The addition of Si in an AlTiN coating, as with Tool A, can reduce coating–substrate adhesion [12,26,28]. For both tools, the presence of surface flaws from droplets (Tool A) and scratches (Tool D) could have also reduced coating adhesion [21,24]. Overall, Tools A and D showed moderate cohesion and poor adhesion with the substrate, suggesting that when exposed to defects or small amounts of cohesive delamination, the tools would be expected to undergo abrupt tool failure.

Throughout the scratch test, Tool B showed very minimal coating delamination, substrate exposure, or cracking. From the scratch pattern in Figure 5b, the coating on Tool B appeared to undergo some buckling, which is typical for thin coatings [26], and perhaps some recovery spallation. The recovery spallation and overall good adhesion performance for Tool B were likely due to the tool’s high H/E value. It has been found that coating adhesion increases with increased H/E [12]. This is because the high ductility and strength of the material allow for some elastic recovery [17,21,26]. Furthermore, Tool B had minimal surface flaws, which may have contributed to good coating adhesion [21,24]. Given the strong cohesion and adhesion capabilities of Tool B, it was expected that this tool would have been able to withstand abrasion and adhesion wear well.

For Tool C, initial cohesive failure began at a very low load. This was expected because of its thick coating. It has been found that thick coatings reduce the critical load due to their high residual stresses [26]. However, it has also been noted that for a thick, hard, brittle coating combined with a ductile substrate, the coating is able to carry a higher load, and spallation can be mitigated if the residual stresses are not too high [26]. Given the scratch trace in Figure 5c and that the coating on Tool C comprised two thick layers, it is likely that both these phenomena occurred. The scratch track showed that Tool C failed by wedging spallation and these spallation marks were more present in the initial stages of delamination. This suggests that the outer Al_2_O_3_ layer was more likely to delaminate. The inner TiCN layer is known to be very hard and abrasion-resistant [10]. As a result, spallation was mitigated and the Lc2 critical load was quite high compared to the Lc1 value.

### 3.3. Cutting Tests

**Figure 6:**
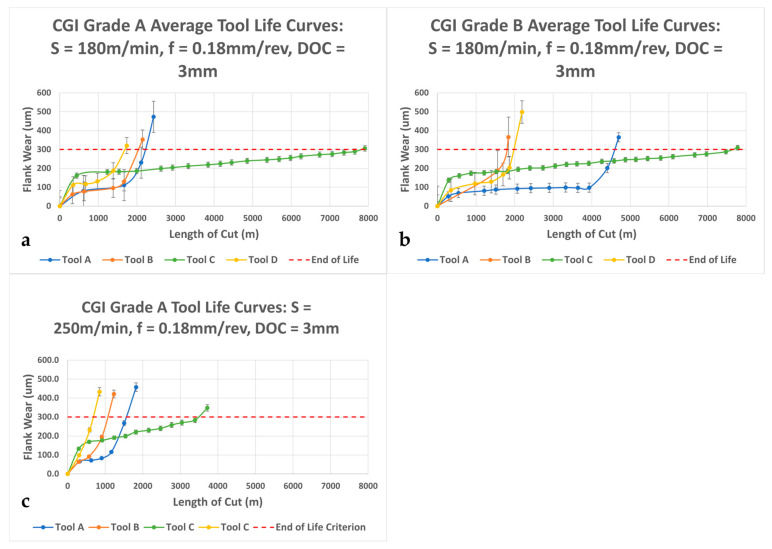
Tool life curves for (**a**) CGI Grade A at S = 180 m/min; (**b**) CGI Grade B at S = 180 m/min; and (**c**) CGI Grade A at S = 250 m/min.

Figure 6 shows the average tool life curves for the CGI Grade A and Grade B cutting tests. The best performing tool was Tool C, which had a high initial wear, but was very stable throughout the remaining tool life for both CGI Grade A and Grade B. It is evident from Table 2 that Tool C’s coating was significantly thicker than those of the other tools. While thick coatings can certainly improve tool life by being better able to withstand high loads, they also introduce drawbacks, such as high residual stresses and material costs, that may outweigh their benefits and make them less suitable for industrial applications [7]. In [19], the author found that AlTiN coating thickness did not impact tool performance outcomes. As such, it is critical to consider Tool C’s coating thickness in conjunction with its other properties to appropriately evaluate and explain its good performance.

Of the four tools, only Tool A showed a notable difference in tool life when machining CGI Grade A and Grade B. The tool life was about 110% longer for Grade B compared to Grade A. The difference in tool life for Tool A between workpiece grades and the lack of difference for the other tools are addressed in conjunction with their wear analyses in Section 3.5.

The large error bars for Tools A, B, and D indicate high variability in tool life between trials, suggesting that they were sensitive to flaws and workpiece inconsistencies. A sensitivity to defects can also lead to abrupt, or very rapid, tool failure, which was reflected in Tools A’s, B’s, and D’s tool life curves. Abrupt tool failure can occur because of poor substrate adhesion, as was seen in the scratch test results (Table 3, Figure 5), but it can also occur when a tool undergoes diffusion wear if the temperature exceeds the activation energy and leads to an exponential increase in wear [29]. To determine each tool’s individual cause for tool failure and how these causes are associated with their properties, wear mechanism analysis should be considered.

When the cutting speed was increased from 180 m/min to 250 m/min, the tool life decreased for all four tools, as shown in Figure 6c. This was expected, as an increase in cutting speed introduces effects such as increased temperatures, shear stresses, and friction. The decrease in tool life was proportional for all tools except Tool A, as those of Tools B, C, and D decreased by 50%, but that of Tool A only decreased by 25%. This suggests that cutting speed was not as influential to Tool A’s behavior as it was for the other tools. For this change in cutting speed, the relative performance rankings of the tools did not change from the baseline 180 m/min speed. However, because Tool A’s decrease in tool life was proportionally lower than those of the other tools, if cutting speed were to continue to increase, Tool A may eventually overtake Tool C as the best performing tool. The specific effects of cutting speed, as well as workpiece microstructural properties, on each tool are analyzed further in the wear mechanism discussion.

**Figure 7:**
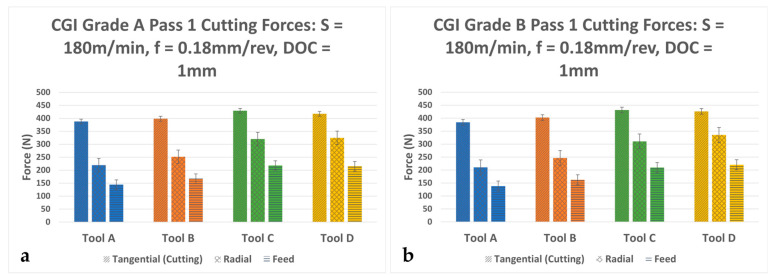
Average Pass 1 cutting forces for (**a**) CGI Grade A and (**b**) CGI Grade B.

Figure 7 shows the Pass 1 cutting forces for the CGI Grade A and CGI Grade B cutting tests. Given the high initial wear of Tool C, it was expected that it would also have the highest cutting forces in the first pass because, with a larger wear landing on the tool, friction between the tool and workpiece increases. Furthermore, Tool C had a very large cutting-edge radius which also increases cutting forces. As cutting-edge radius increases, the tool is not only shearing the workpiece material but also plowing it. This requires large amounts of energy and thus increases cutting forces. On the other hand, because the wear on Tool C was stable, a minimal increase in forces can be expected throughout the rest of the tool life compared to large increases in cutting force for the other coatings. Tool C’s stable behavior is further explored during the wear analysis in Section 3.5.3. Tool A exhibited the best cutting force performance out of the four tools. However, the margin for the increase in performances was drastically different between tool life and cutting forces. Tool A had a 10% higher performance in tangential force over Tool C for CGI Grade A, but a 250% lower performance in tool life. Similarly, Tool A had a 12% higher performance in tangential force over Tool C for CGI Grade B, but a 70% lower performance in tool life.

### 3.4. Chip Analysis

**Figure 8:**
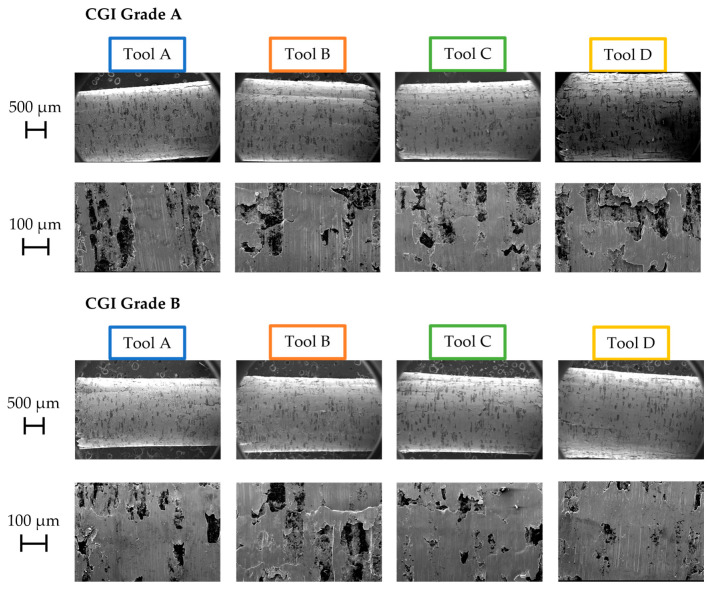
Undersurface of Pass 1 chips.

**Figure 9:**
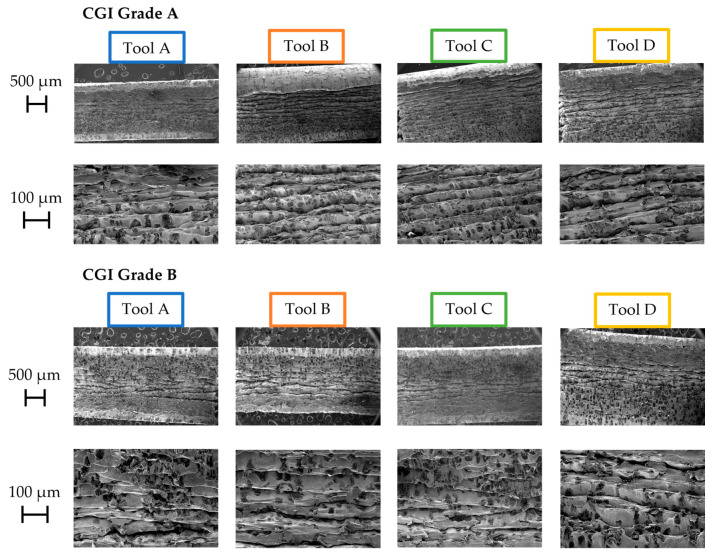
Shear bands of Pass 1 chips.

Figure 8 and Figure 9 show the Pass 1 chips collected for each tool–workpiece combination. The three factors of chip morphology examined to characterize tribological properties were chip curling, slipping patterns on the undersurface of the chips, and shear bands. Note that the black marks seen on the chips in the figures are graphite.

Although chip curling was somewhat challenging to compare for this workpiece given that its brittle nature created very small chips, Tool A and Tool C appeared to have a higher degree of curling compared to the other tools, and Tool D had the lowest. Chip curling indicates that the workpiece material is able to flow rapidly across the rake face, creating a difference in deformation rates between the sides of the chip and causing the chip to form a curl pattern [19]. For material to be able to flow across the rake face at a high velocity, friction between the chip and the tool must be minimized [17,19,30].

In terms of slipping patterns on the underside of the chips, similar trends were seen, with Tools A and C exhibiting continuous slipping patterns and smooth chip undersurfaces, and Tools B and D showing some tears and discontinuities across the slipping patterns (Figure 8). Tears and discontinuities are caused by the “stick and slip” of the chips to the rake face [19]. Under poor tribological conditions, the chip will stick to the rake face until a build-up of material and pressure cause it to be torn away [19,30].

As with the slipping patterns and chip curling, Tools A and C tended to have regularly spaced and unbroken shear bands, while Tools B and D showed more discontinuous shear bands (Figure 9). Regular shear bands indicate that plastic deformation is occurring uniformly across the rake face and that there is an absence of “stick and slip” [17]. If one area of the chip is adhered to the rake face but the rest of the chip continues to slide, the shear band will be broken, and the width of the shear band may vary along its length. Furthermore, if the entire chip is undergoing “stick and slip”, the thicknesses of the shear bands will be inconsistent overall. Therefore, uniform shear band formation is associated with good tribological performance.

With all three chip morphology characteristics considered collectively, Tool A had the best friction performance, followed by Tool C, Tool B, and Tool D, in that order. Tool A’s superior friction performance can be attributed to its excellent surface finish and the presence of amorphous carbon at its surface, which has self-lubricating properties [12]. However, given its low hardness and toughness, it could be expected that these optimal surface conditions would not remain as tool wear progressed.

Given Tool B’s superior toughness and surface finish compared to Tool C, it may have been expected that it would exhibit better tribological properties out of the two tools. In this case, Tool C’s moderately good friction performance may be attributed to its thick outer Al_2_O_3_ coating. Al_2_O_3_ can act as a solid lubricant that inhibits surface finish deterioration [17]. Tool B contains some Al, which oxidizes to form Al_2_O_3_ upon machining, but in much lower amounts compared to Tool C, as seen in the EDS scan shown in Figure 2. Therefore, Tool B’s surface finish would likely have deteriorated much more rapidly than that of Tool A, even at the beginning of the tool’s life, and subsequently resulted in a worse friction performance. Due to Tool C’s thick, thermally stable Al_2_O_3_ outer layer, its friction performance likely would have been relatively stable throughout the rest of its life.

Although Tool D had the highest hardness of the four tools, it also had the worst friction performance. One reason for this is the thermal softening of the coating under cutting temperatures which can lead to rapid deterioration of the tool’s surface finish [17]. Tool D had a lower Al content in its coating compared to the other tools (see Figure 2) and thus was lacking thermal protection from Al_2_O_3_ to maintain its initial hardness value. Substrate effects would have also increased Tool D’s thermal sensitivity, as its grain size and binder content led to poor thermal properties. These effects would have increased throughout the rest of the cutting test as temperatures continued to rise.

### 3.5. Wear Analysis

#### 3.5.1. Tool A

**Figure 10:**
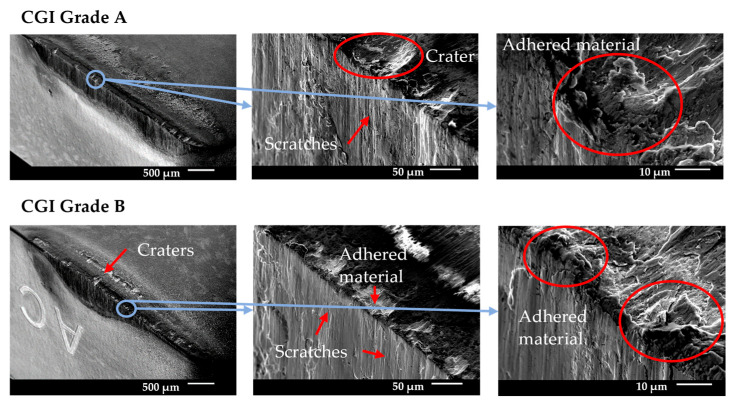
Tool A end-of-life SEM images.

**Figure 11:**
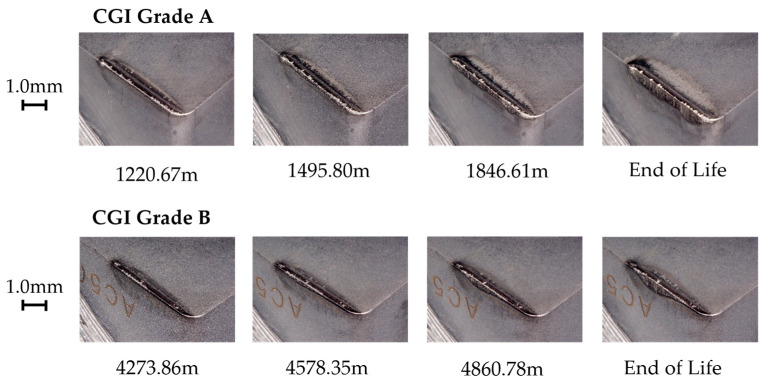
Tool A rapid wear progression.

Tool A showed minimal wear for both grades of CGI until the rapid wear stage. Figure 10 shows that wear occurred primarily on the flank face and there was minimal cratering, built-up edge, or chipping, particularly compared to the large amount of scratching. Figure 11 shows that areas of high flank wear originated in areas with high adhesion on the rake face for both workpieces. Therefore, Tool A seemed to be highly susceptible to flaws on the tool. This can also be seen by the high degree of variation in performance between trials in Figure 6. Tool A’s flaw sensitivity can be attributed to poor substrate toughness, poor coating adhesion, and thin coating thickness [7]. Because the flaws introduced to Tool A were a result of adhesion, Tool A’s good tribological properties (see chip analysis) and cohesively strong coating likely helped mitigate its failure and led to its better performance over Tools B and D [7,9].

For CGI Grade A, EDS data showed that there was more adhered material, and it was distributed more continuously than for Grade B, with approximately 14% and 6% adhered Fe, respectively. This suggests that machining CGI Grade B led to more chipping than Grade A. The differences in adhesion behavior between grades may explain the improved tool life for CGI Grade B seen in Figure 6. For workpiece material to adhere to a surface, the cohesive strength within the workpiece must be overcome by the adhesive strength of the junction formed with the tool material [27]. The adhered material can then come loose in two ways: either the adhesive strength of the junction fails, which reverses the adhesion, or the cohesive bonds within the tool are overcome, which causes chipping [27]. Note that for coated tools, chipping can occur through both cohesive failure of bulk tool material or adhesive failure in the form of coating delamination. If CGI Grade B is cohesively very strong due to its increased nodularity, it is less likely to adhere to the tool surface than Grade A, but more likely to cause chipping when it does [27]. Overall, this can result in less adhesion-induced flaws for CGI Grade B. Since Tool A was sensitive to flaws in the tool, including those introduced by adhered material, it follows that tool wear would accelerate more rapidly for CGI Grade A, where more flaws were introduced.

Despite the role of adhesion in Tool A’s wear acceleration, the overall failure mechanism was abrasion. Given the differences in adhesion behavior between the workpieces, it stands to reason that any other introduction of flaws would lead to similar rapid abrasion wear, thereby supporting the conclusion that abrasion is dominant. Furthermore, Tool A had the best friction performance of the four tools, as is evident from its low cutting forces, continuous slipping pattern on the chips, and significant chip curling. The adhesion behavior of Tool A was not the most dominant factor leading to tool failure, but it can highlight the impact of the extreme cutting conditions in the tests. The nature of tool failure for Tool A was such that surface flaws did not need to be large to have a significant impact on tool life. Therefore, Tool A is the only tool for which small changes in workpiece properties resulted in differences in tool performance, regardless of the extreme cutting conditions.

The addition of Si in Tool A’s coating likely had several conflicting implications on its tool life and wear behavior. On one hand, the presence of Si in the coating is predicted to have contributed to its poor substrate adhesion, which was one of the root causes of its flaw sensitivity [12,26,28]. On the other hand, the thermal stability of Si-containing AlTiN coatings would have mitigated diffusion wear and therefore reduced the introduction of additional surface flaws [11,12,28,31]. Overall, the cutting test results showed that when machining CGI Grade B, for which the drawbacks of a Si-containing AlTiN coating were less significant, the addition of Si to a typical AlTiN coating could be beneficial. However, when workpiece properties and cutting conditions highlight the weaknesses of adding Si to the coating, such as when machining CGI Grade A, Si-containing AlTiN coatings would be a poor choice.

#### 3.5.2. Tool B

**Figure 12:**
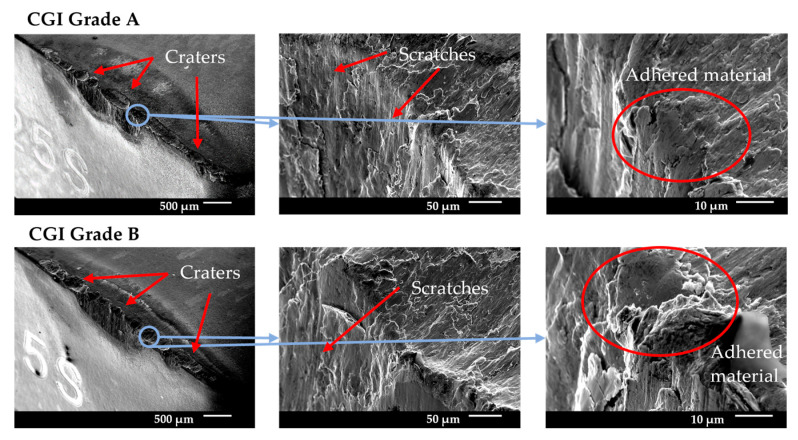
Tool B end-of-life SEM images.

**Figure 13:**
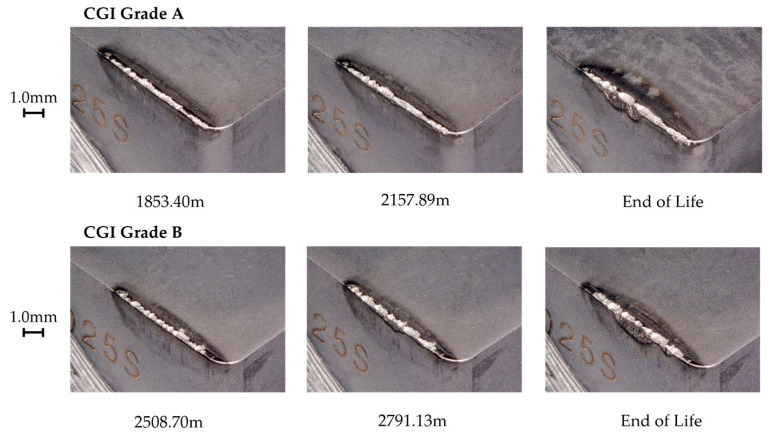
Tool B rapid wear progression.

Tool B showed minimal wear for both grades of CGI until the rapid wear stage. Large craters were formed on the rake face and flank wear was localized in areas with more significant cratering, as can be seen by the SEM images in Figure 12. The images of Tool B’s rapid wear progression in Figure 13 highlight the negligible wear on the flank face while significant wear was already visible on the rake face, suggesting that flank wear was a response to the cratering and that diffusion was the dominant wear mechanism. It has been reported that the inclusion of Si in AlTiN coatings improves the thermal stability of the tool which is at odds with the observed results of Tool B’s cutting tests [11,12,28,31]. As such, the addition of Si to the AlTiN coating had a negligible effect on tool life and performance for Tool B. Rather, it was the high heat generated from its large cutting edge and high hardness that made Tool B susceptible to thermally activated wear mechanisms. In addition, the high Co content coupled with a relatively fine grain size in Tool B’s substrate further reduced its thermal stability. Given its thin coating thickness, the substrate was more readily exposed, which would have also accelerated diffusion wear [7,9].

Given Tool B’s sensitivity to thermally activated processes, any small changes that may have otherwise improved tool life had no impact, because the extreme cutting conditions likely ensured that the temperature was always above the activation energy for diffusion. In this case, the increased nodularity of CGI Grade B led to the expectation that this grade would have higher cutting temperatures than Grade A, leading to a shorter tool life. However, the differences in tool life were negligible, thus demonstrating that the effects of the extreme cutting conditions were dominant over the changes in workpiece properties.

#### 3.5.3. Tool C

**Figure 14:**
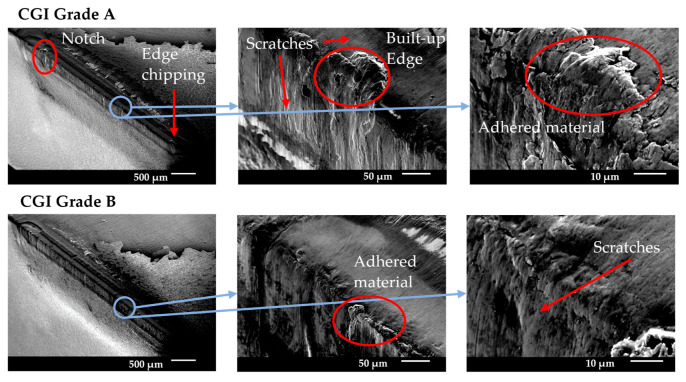
Tool C end-of-life SEM images.

**Figure 15:**
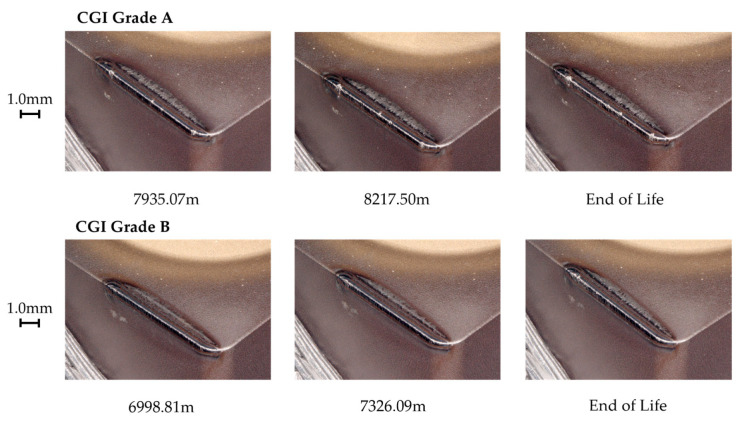
Tool C rapid wear progression.

Tool C showed a relatively high amount of flank wear after the first pass, but very minimal change in wear until the rapid wear stage. For both CGI Grade A and Grade B, Tool C had a minimal built-up edge and wear occurred primarily on the flank face. The SEM images in Figure 14 show some notching, edge chipping, and small amounts of built-up edge, but the cutting edge was overall intact, indicating that defects did not compromise the integrity of the cutting edge. The main wear pattern seen for Tool C was large amounts of scratching, suggesting that abrasion was an important phenomenon in tool failure. On the other hand, Tool C showed a strong resistance to oxidation and diffusion due to the negligible cratering on the rake face and low evidence of oxidation in the EDS scan. The rapid wear images in Figure 15 show that flank wear was extremely uniform, even in areas with adhesion or in the presence of other flaws, such as notches. Thus, the dominant wear mechanism for Tool C was abrasion because of its stable wear performance, evidence of abrasive marks, and minimal evidence for other failure mechanisms. For Tool C, the change in workpiece properties had a negligible impact on tool life and failure mechanisms.

Tool C’s long tool life, stable wear, and low sensitivity to changes in workpiece microstructure were likely a result of several reasons. Tool C had a very thick coating, so a large portion of the cutting-edge strength was sustained by the stronger coating, rather than the substrate [7,9]. Furthermore, the thick inner TiCN coating layer showed strong adhesion to the substrate, which mitigated substrate exposure and allowed wear to remain stable, similarly to its results in the scratch test [10]. These two properties likely contributed to Tool C’s resistance to wear acceleration despite the presence of notching and adhesion. Strong adhesion to the substrate mitigates flaking or chipping of the coating, even around areas with flaws, such as notches [26]. This effect would have been increased by the thickness of the coating, which makes gross delamination less likely to occur, especially for very hard coatings as was the case with the TiCN coating on Tool C [26]. Furthermore, due to the thickness of the coating, a greater volume of tool material had to be worn away to expose the substrate and cause the tool to enter the rapid wear stage. Therefore, even though notching and adhesion were present, these flaws did not accelerate tool wear, as their effects were both minimized and delayed. The thick outer Al_2_O_3_ layer would have mitigated diffusion and helped to reduce adhesion and friction effects, but this was likely less critical to its overall performance than the influence of the TiCN layer [32]. Because Tool C’s wear landing and cutting-edge integrity remained unaffected by chipping and notching, an increase in adhesion and thermally activated wear as a result of a thinner Al_2_O_3_ layer likely would not have had a significant impact on overall tool life and tool performance. In addition, Tool C’s good thermal properties in its substrate would also help protect the tool in the absence or reduction of Al_2_O_3_ in the coating [25]. As a result, it was likely Tool C’s thick TiCN coating layer, specifically, that helped protect the substrate and cutting edge under the extreme cutting conditions and new stresses introduced with CGI Grade B.

#### 3.5.4. Tool D

**Figure 16:**
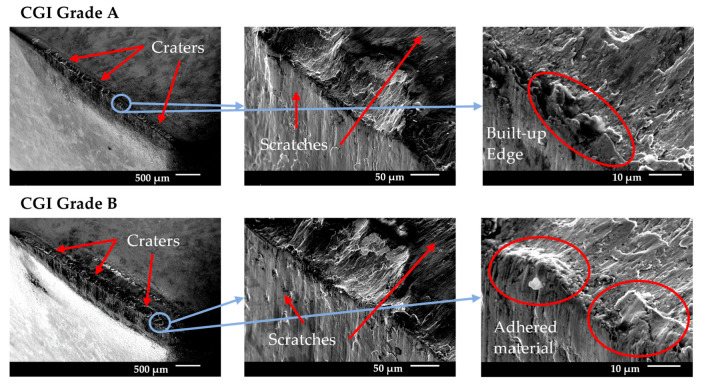
Tool D end-of-life SEM images.

**Figure 17:**
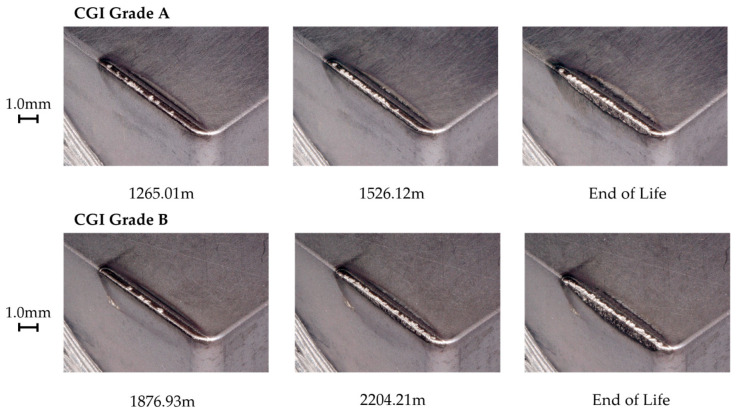
Tool D rapid wear progression.

Tool D showed uniform flank wear throughout the cutting tests for both CGI Grade A and Grade B. Wear occurred both on the flank and rake faces. The SEM images in Figure 16 show significant scratching on both faces, as well as moderate cratering on the rake face and adhered material on the wear landings. The rapid wear progression images in Figure 17 show that abrasion wear was not localized to areas of high adhesion. Furthermore, the initial flank wear on Tool D was quite high compared to the other tools, especially considering its small cutting-edge radius and low surface roughness. Therefore, the dominant wear mechanism for Tool D was likely abrasion. Despite exhibiting a significantly higher toughness than the other tools, low surface roughness, and high coating hardness, Tool D failed very rapidly and had the worst tool life out of all the tools.

Hard and tough tools are typically expected to perform well when machining hard-to-cut materials such as CGI [2,6,9]. However, Tool D had a very poor elastic deformation resistance, as seen by its H/E value in Table 3. This means that small deformations resulted in very large stresses that the tool material was unable to sustain, despite a high hardness. This effect would have been magnified by Tool D’s poor substrate adhesion and thin coating, which decreased the load Tool D was able to sustain [17,32]. A high tool hardness would have also increased cutting temperatures that may have led to a greater degree of thermally activated wear, but because the dominant wear mechanism for Tool D was abrasion, the thermal effects of Tool D’s hard coating were less significant in this application.

Any apparent improvement in tool life between CGI Grade A and Grade B was not statistically significant and it can be assumed there was no difference in performance between workpieces. This is likely because Tool D’s inability to withstand stresses induced by the cutting conditions was so great that the change in workpiece properties was not sufficient to have an effect on tool performance.

#### 3.5.5. Overall Tool Comparison and Selection

From the tool life tests and wear analysis, it is evident that Tool C was the best performing tool for both workpieces by a very large margin. When comparing Tool C’s performance for CGI Grade A and CGI Grade B, it appears that the magnitude of the workpiece effects was proportionally insignificant given the dominant performance of Tool C. In other words, a much more significant change would be necessary to elicit a change in tool performance. An example of such a change is the increase in cutting speed from 180 m/min to 250 m/min, shown in Figure 6. At the higher speed, the tool life for Tool C was significantly reduced. It could be deduced that cutting speed was the dominant characteristic driving tool behavior or tool life. However, we would then expect to see proportional results with the other tools, but while all other tools had a shorter life by about 50% at 250 m/min, Tool A’s life was only reduced by approximately 25%. It is more likely that each system defined by a workpiece, tool, and cutting conditions has properties with varying degrees of impact on tool behavior. Then, if the property changed is one of lesser importance, a larger change would be necessary to have an effect on tool performance, while a high impact property would only necessitate a small change to affect tool performance.

For the purposes of tool selection, it is important to understand how the combination of properties and conditions shape the behavior of the tool, and which tool properties can most effectively improve tool performance. In this study, the cutting conditions had a high impact on tool behavior. Therefore, only extreme tool properties, such as the thick coating on Tool C, or highly impactful changes, such as the change in adhesion behavior between workpieces for Tool A, could lead to an improvement in tool performance. However, despite the improved performance for Tool A between CGI Grade A and Grade B, the most significant tool parameter maximizing tool life under extreme cutting conditions was the thickness of the coating on Tool C. As such, if a tool was selected purely based on its tool life and wear performance, there would be no significant differences in the tool selection process when machining different grades of CGI.

Cutting test results showed that the addition of Si to typical AlTiN coatings can be effective in improving tool life under the right conditions. Si-specific tool properties only increased tool life when machining CGI Grade B with Tool A, but decreased tool life when machining CGI Grade A with Tool A and had a negligible effect on tool life when using Tool B. Hence, it cannot be conclusively claimed that Si-containing AlTiN coatings improve cutting performance when machining CGI. Instead, these results highlight the importance of considering all system properties and parameters collectively in the tool selection process, and that only with consideration and optimization of those parameters, doping an AlTiN coating with Si can be used as an effective strategy to increase tool life.

Practically, financial aspects must be considered when selecting a tool. Given its thick coating, Tool C would be a more expensive option than Tool A. In addition to the costs incurred from more material, the CVD process is more expensive than PVD. Furthermore, thick coatings are typically less common in industry, making tools with thick coatings more likely to be expensive as they are less standard. If the higher tool costs for Tool C would be offset by the reduction in machine downtime and reduction in tool replacement costs from the increased tool life, Tool C would be the best tool choice. Otherwise, Tool A may be a better option, especially when machining CGI Grade B, for which it showed an improved tool life. These financial implications differ between manufacturers, so a deep understanding of the interactions between different factors in the machining process is necessary to consolidate financial considerations with tool performance and make an informed decision.

## 4. Conclusions

The cutting performances of four commercially available tools were compared for two different grades of CGI under extreme cutting conditions.

The following conclusions were reached in this study:

i. For all tools with the exception of Tool A, which had a thin PVD coating, the tool life and wear mechanisms remained unchanged between workpieces. Tool A’s life increased when machining the higher-nodularity CGI Grade B due to the reduction in surface flaws from adhesion.
ii. The addition of Si to AlTiN coatings improved tool life when machining CGI Grade B with Tool A, but either reduced tool life or had no impact for the other cutting tests. Therefore, AlTiSiN coatings will only improve tool life when machining CGI under specific conditions.
iii. Tool C was the only tool with a thick CVD coating, and it outperformed the other tools significantly for both workpieces. Thus, for extreme cutting conditions, highly impactful tool properties such as a thick coating are much more significant to the tool selection process than small microstructural changes to the workpiece.
iv. To make the best tool selection, financial considerations must be coupled with a comprehensive understanding of tool, workpiece, and cutting condition interactions.

This research demonstrates that system properties, including cutting parameters and tool/workpiece characteristics, must be considered collectively to evaluate tool behavior. An approach to evaluate property interactions should be explored in future work. Such work could include studies on coating development. This research was focused on commercially developed tooling. However, being able to vary deposition parameters, chemical composition, and microstructure, among other properties, to create custom tooling could provide further insight into the interactions of different parameters. In doing so, the design process of unique and focused solutions for a specific machining application could be streamlined.

## Figures and Tables

**Table 1:** Literature summary of tool coating composition and depth of cut for given cutting parameters.

		Low Speed	Moderate Speed	High Speed
		≤200 m/min	250–300 m/min	≥400 m/min
Low Feed	0.04–0.05 mm/rev		[4]: TiCN/Al_2_O_3_, TiCN/Al_2_O_3_/TiN; 2 mm	[3]: PCBN; 1 mm
High Feed	0.2–0.5 mm/rev	[14]: TiN; 0.15 mm, 0.2 mm [1]: PCBN; 0.2 mm	[7]: TiAlN; 0.25 mm [1]: PCBN; 0.15 mm, 0.2 mm [9]: TiAlN; 0.25 mm [10]: TiCN/Al_2_O_3_/TiN; 0.2 mm	[1]: PCBN; 0.15 mm, 0.2 mm [8]: PCBN

**Table 2:** Summary of tool geometric properties.

Tool	Coating Thickness (µm)	Cutting-Edge Radius (µm)	Sa (µm)
A	2.27 ± 0.02	36.700	0.314 ± 0.016
B	2.33 ± 0.02	45.004	0.320 ± 0.016
C	14.21 ± 0.14/6.58 ± 0.06	46.397	0.338 ± 0.017
D	2.63 ± 0.02	36.495	0.266 ± 0.013

**Table 3:** Summary of coating mechanical properties.

Tool	Hardness, H (GPa)	Elastic Modulus, E (GPa)	H/E	Toughness (N/μm)	Lc1 (N)	Lc2 (N)
A	26.64 ± 4.6	341.7 ± 66.5	0.078	1.74 ± 0.02	50.34	60.67
B	34.27 ± 8.8	363.0 ± 72.4	0.094	2.47 ± 0.02	66.97	89.28
C	30.32 ± 6.9	375.5 ± 84.6	0.081	2.21 ± 0.02	28.62	71.11
D	35.75 ± 5.2	481.4 ± 74.9	0.074	6.37 ± 0.06	54.37	70.27

## Data Availability

Data are contained within the article.
